# Chile’s dilemma: how to reinsert scientists trained abroad

**DOI:** 10.12688/f1000research.5287.1

**Published:** 2014-09-18

**Authors:** Alexia Nunez-Parra, Maria-Paz Ramos

**Affiliations:** 1 Department of Cell and Developmental Biology, Rocky Mountain Taste and Smell Center and Neuroscience Program, University of Colorado Medical School, Aurora, 80045, USA; 2 Department of Genetics, Albert Einstein College of Medicine, New York, 10461, USA

## Abstract

Chile is recognized worldwide as an emergent economy, with a great power in natural resource exploitation. Nonetheless, despite being one of the most developed countries in Latin America, Chile imports most of the knowledge and technology necessary to drive innovation in the country. The tight budget that the Chilean government assigned to research and development and the absence of a long-term scientific agenda contributed to a limited supply of scientists over the years. In an effort to reverse this scenario, Chile has created several fellowships, such as the Becas Chile Program (BCP) to encourage new generations to pursue graduate studies to ultimately advance research and development *in situ*. More than 6000 fellows are now being trained abroad, accumulating an incredible potential to transform the Chilean scientific environment as we know it.  Chile now faces a greater challenge: it has to offer infrastructure and job openings to the highly skilled professionals in whom it invested. Unfortunately no clear public policies to address this situation have been developed, partially due to the lack of a dedicated institution, such as a Ministry for Science and Technology which could focalize the necessary efforts to promote such policies. Therefore, in the meantime, Chilean scientist have been motivated to create different organizations, such as, *Mas Ciencia para Chile *and *Nexos Chile-USA*, to promote constructive discussion of the policies that could be implemented to improve the Chilean scientific situation. We hope that these and other organizations have a real impact on the generation of scientific guidelines that will finally contribute to the development of the country.

## Commentary

Chile is catalogued worldwide as one of the most stable countries in the Latin-American region. It is seen as an example of a growing economy, experiencing an average gross domestic product (GDP) growth rate of 5.3% in the last four years ( World Development Indicators, World Bank data). This economic stability continuously attracts several international corporations that settle and open new branches in its territory. Chile is referred to as a flourishing nation with an intrinsic desire to innovate and reach international technological standards. Moreover, in 2010 Chile became the first South American country to join the Organization for Economic Cooperation and Development (OECD), recognizing Chile’s prosperous economy and strong democratic union.

The Chilean economy is fundamentally sustained by natural resources, such as copper extraction, as well as fruit production and exportation. Therefore, the next logical step would be to invest in novel technology to expand the already established industries, and furthermore, define new strategies to generate sustainable growth. Paradoxically, despite its economic power, only 0.42% of the GDP is assigned to Research and Development (R&D) in Chile ( Research and development expenditure, World Bank data). Moreover, the funds for the formation of advance human capital have been historically low. As an attempt to reverse that situation, Chile created the Republic President Fellowship program in 1981. The tight budget, however, considered only two years of specialization, excluding the possibility of pursuing longer programs, such as a PhD. In addition to the governmental program, international organizations, such as Fulbright, collaborated with the Chilean National Commission for Science and Technology (CONICYT) to grant fellowships to Chilean students to study abroad. This program, known as the Equal Opportunities Fellowship, was discontinued due to the great monetary investment that it involved.

In an effort to promote the development of the sciences, Chile set a long term goal to join the knowledge society in which knowledge is generated and shared to ultimately improve the human condition through innovation and technology. As a first approach, and in order to inform the community about the importance of science, CONICYT created several programs, such as the *Explora* projects, to teach middle school children science in a didactic way, or *La Ciencia nos cambia la Vida,* mini documentaries about scientific developments created in Chile that impact the quality of life of its own population. Moreover, as part of the plan to reach this ambitious goal, in 2008 the Chilean government created the Becas Chile Program (BCP). This fellowship, under the direction of CONICYT, aims to increase the opportunities of continuing education in foreign countries, coordinates the national fellowship program, and enhance international cooperation. Seizing the opportunity, Chilean students and professionals applied for the fellowships and massively enrolled in graduate programs (masters and PhDs) and postdoctoral programs in several countries worldwide. The number of fellows jumped from less than 200 annually before the creation of the BCP, to more than 1200 within the first year of the program implementation (Statistics indicators, Becas CONICYT International). After six years of operation, 6,042 fellowships have been granted in order to pursue masters and doctorates abroad ( Statistic indicators, Becas Chile).

With the implementation of BCP, an important and historical advancement in the formation of human capital has been made. Chile will have for the first time a number of highly specialized professionals to advance science, technology and innovation. However, this explosive increase in the number of available fellowships carried enormous consequences. The lack of an institution controlling the necessary policies (such as a dedicated ministry) focused on reintegrating the highly skilled professionals trained abroad back into the Chilean academic and industrial system, has become evident. This problem was already detected and presented in the conclusions of the revision of the BCP performed by the OECD in partnership with the World Bank in 2010 ^1^. In this review, they state, *“more purposeful efforts will be needed to maximize the re-integration of BCP graduates, especially in several fields where the country has limited scale and scope”.* Including scientists studying abroad by other means (i.e. supported by international grants or academic programs), we estimate that the number of scientists that will have to be reinserted in Chile adds several hundreds more per year to the already 6,000+ BCP fellows. The problem gets aggravated by the fact that the fellowships stipulate that the recipients have to return to Chile after the fellowships end for twice the number of years they were studying abroad. If they do not return, they have to pay back the investment granted for their education. It is expected that Chile would reap the rewards of investing in human capital training and prevent brain drain. Nevertheless, the country is unfortunately not fully prepared to benefit from all these professionals as there are not enough jobs available nor the infrastructure required to support them.

In order to improve that situation, some efforts have been taken to offer opportunities and take advantage of the new experts. CONICYT has recently created fellowships and grants to attract professionals that are residing abroad, but the number of fellowships available is still limited, reaching no more than 25 per year ( Attraction and Insertion of Advanced Human Capital Program, CONICYT).

The weaknesses of the scientific system in Chile that are leading to sparse permanent job openings have been discussed by several institutions and organizations. Some of the most recurrent ones mentioned are Chile’s lack of governance and scientific support and the reduced involvement of the private sector in the development of science and technology ^2^. The planning, development and implementation of public policies in science occurs in different ministries and institutions to the detriment of well-planned long-term policies and scientific agenda ( Final report 2013, National Presidential Commission). Without clear guidelines, it seems difficult to create a stable scientific system fed by basic and applied research that could lead to economic growth in the country. This is in contrast to most other OECD countries that contain a Ministry of Science and Technology or similar institution that helps to converge efforts for advancement in science, technology and innovation. Moreover, even though the budget assigned for research and development has been doubled since 2009, reaching 1.037 billion dollars in 2013 ( CONICYT Annual Report 2013), it is still considered very limited considering that it has been maintained at around 0.4% GDP for the last five years. This situation contrasts with the percentage that others countries in the region designate to science, for instance Argentina and Brazil contribute approximately 1% of their GDP. In addition, as opposed to what occurs in more developed countries, the interest of the private sector in investing into scientific research is minimal. Chile does not have a system that provides incentives to private fundraising foundations, nor companies to generate technology and innovation *in situ*.

Nevertheless, despite the lack of considerable funding, the quality of science that is produced in Chile is one of the best in the region, being the country with the highest ranking in terms of number of ISI publications per hundred million dollars invested and per number of inhabitants ( CONICYT and RYCIT). The high quality science performed in Chile and the fact that thousands of highly prepared scientists will populate the country in the next years creates incredible potential for the prospect of Chilean science. The current scenario is ideal for Chile to make the jump forward by taking advantage of the situation and positioning Chilean science as one of the top in the world. Today is the moment to increase the scientific budget and define a clear strategy for the reinsertion of the thousands of professionals that will go back to Chile to implement the state-of-the-art technologies and knowledge acquired abroad. At the same time, new buildings and laboratory space specially designed to meet the needs of the researchers are required and badly needed.

### What to do in the meantime?

The lack of an institution in charge of the scientific development will hardly change in the short-term. President Michelle Bachelet has not disclosed a clear agenda to promote public policies in science. While the Senate has sent a request to create the Ministry for Science and Technology, the necessary laws may take several years to be promoted, especially since the efforts of the current administration are mainly focused in the popular concern of transforming the educational and tax systems ( Program of Government, Chilean Government). Even though a general feeling regarding the importance of the Chilean scientist abroad has started to grow, researchers observe with concern the lack of policy implementations needed for scientific development in Chile and the real possibilities of finding interesting opportunities back home.

In such a scenario, a group of scientists in Chile started a social movement to generate a productive discussion about the importance of science and technology in the development of a country. The organization they created at the end of 2010, called *Mas Ciencia para Chile,* is today a foundation that aims to promote the debate about the importance of developing scientific policies in Chile. In parallel and with a similar perspective, Chilean researchers residing abroad have realized the necessity of contributing in the meantime, and eventually in parallel with the appropriate authorities, to promote the reinsertion of their newly advance trained group into the country. As an attempt to discuss such strategies and gather the large number of scientists working and studying in the United States, Nexos Chile-USA was founded in 2010 ( www.nexoschileusa.org). Nexos aims to communicate and develop collaborations between its members, and more importantly, to promote interaction between Chilean scientists in the USA and their peers in Chile. In addition, Nexos Chile-USA has become a platform to inform its members about different paths for the reinsertion after their training abroad, and to offer options for alternative careers outside academia. Nexos has created multiple tools to communicate with its community: from monthly newsletters about grant or job opportunities, interviews with prestigious scientists who give advice on how to build a scientific career, to documents guiding PhD applications and life in the U.S.

Possibly, the most valuable instrument that Nexos Chile-USA has implemented to pursue its aims is its annual meeting, which in close collaboration with the Embassy of Chile in Washington D.C., gathers around 150 scientists from all around the country. This annual meeting is an opportunity to listen to scientific talks of great value from other Chilean scientists and workshops aimed at facilitating professional careers, as well as a tool to discuss scientific polices with relevant players of main funding agencies in Chile. Finalizing this year’s meeting at in October, the organizing committee plans to elaborate and disseminate a document with the predominant topics discussed during the meeting and the round table: “Chilean Science: An attractive pole for international collaboration?” including the perspective the community abroad has about the situation in Chile. With such a document, Nexos Chile-USA aims to actively participate in the discussion about the necessary political and scientific infrastructure that will help the advance of science in Chile, in addition to stimulating other organizations and institutions to provide their own novel ideas about such an important topic.

The importance of organizing the Chilean scientific community abroad has been considered in, and spread to, multiple countries, where professionals have created organizations similar to Nexos Chile-USA (including REDICEC in Canada, Red INVECA in Germany, REUK and RedICE in the UK, Ech Francia in France, RedInche in Spain, CREGA in Australia, RIECH and Becpass, among others). These different entities have met multiple times to discuss the necessity of contributing to the discussion of public policies to promote science in Chile and to facilitate a better understanding between the government and the scientific community. We hope that in the near future Nexos Chile-USA, together with these other organizations, will contribute to the development of the country.
