# Supplementary material for: Chile’s dilemma: how to reinsert scientists trained abroad
Source: F1000Res. 2014 Sep 18;3:225. [Version 1] doi: 10.12688/f1000research.5287.1 (PMC4184291; doi:10.12688/f1000research.5287.1)
Supplement: Peer review history

# Peer review: Referee response for version 1

**DOI:** 10.5256/f1000research.5638.r6176

**Published:** 2014-09-22

**Authors:** Gabriel Leon

**Affiliations:** 1 Department of Biological Science, Universidad Andrés Bello, Santiago, Chile

This Opinion Article addresses an important issue that needs to be answered by the Chilean Government: how to reinsert the young scientists trained abroad. In the past years, the Chilean Government has developed international training programs but little has been done in order to generate infrastructure and job openings for those highly trained scientists. This article describes how the scientists are organizing and pushing in order to generate policies that address this problem and others related to the Chilean scientific development, as the dependence on the exploitation of natural resources and how to transform the economy to a knowledge-based one. 

**Minor revisions:**

1. "...with a great power in natural resource exploitation". Change power to emphasis.
2. "Finalizing this year’s meeting at in October", delete "at".
3. Even when this Opinion Article is related to the problem of the reinsertion of scientists trained abroad, is necessary to include in this analysis the number of scientist that were trained in Chile. They are additive to this scenario. 
4. "ANP and MPR both helped to design and write the manuscript..." change to "ANP and MPR wrote and edited the manuscript..."

I have read this submission. I believe that I have an appropriate level of expertise to confirm that it is of an acceptable scientific standard.

# Peer review: Referee response for version 1

**DOI:** 10.5256/f1000research.5638.r6175

**Published:** 2014-09-22

**Authors:** Jorge Babul

**Affiliations:** 1 Department of Biology, University of Chile, Santiago, Chile

This Commentary addresses an important problem for the scientific development of Chile: the training of highly specialized professionals in foreign countries and their reintegration to the Chilean academic and industrial world. The authors describe the Chilean efforts to promote the advancement of science, the weaknesses of the scholarship system, the lack of governance and support, and how scientists in Chile and abroad are getting organized in order to develop scientific policies to solve these problems.

There are a few minor revisions that should be made:

1. Number of fellowships. *It should include separately masters, PhDs and postdoctoral fellowships (only the total number, 6,042, is given).*
2. "[...] partnership with the World Bank in 20101." *Correct year 2010?*
3. "The weaknesses of the scientific system in Chile that are leading to sparse permanent job openings have been discussed by several institutions and organizations." *Mention should be given to the main ones.*
4. "[...] development of science and technology2." *Delete 2.*
5. "[...] at around 0.4% GDP for the last five years." *The number of years should be revised (I would say for the last 15 years). Before Chile joined the OECD, the budget was 0.7% GDP; afterwards it had to be recalculated according to OECD rules, giving 0.4%.*
6. "Chile does not have a system that provides incentives to private fundraising foundations, nor companies to generate technology and innovation in situ." *It has an incentive system for cooperation between industries and research institutions, the so called “I+D Law”.*
7. "[...] positioning Chilean science as one of the top in the world." *This is overstated.*
8. "[...] for the reinsertion of the thousands of professionals that will go back to Chile." *Although this commentary is about reinsertion of professionals that were trained abroad, the number of PhD students that are trained through the national program of scholarships should also be considered; they are part of the problem. Compare the number of PhD scholarships/students with national and international scholarships, for example.*
9. "[...] this year’s meeting at in October." *Delete at.*
10. "[...] will help the advance of science in Chile." *advancement*?

I have read this submission. I believe that I have an appropriate level of expertise to confirm that it is of an acceptable scientific standard.
